# Identification of Gut Microbiome Metabolites via Network Pharmacology Analysis in Treating Alcoholic Liver Disease

**DOI:** 10.3390/cimb44070224

**Published:** 2022-07-19

**Authors:** Ki-Kwang Oh, Ye-Rin Choi, Haripriya Gupta, Raja Ganesan, Satya Priya Sharma, Sung-Min Won, Jin-Ju Jeong, Su-Been Lee, Min-Gi Cha, Goo-Hyun Kwon, Dong-Joon Kim, Ki-Tae Suk

**Affiliations:** Institute for Liver and Digestive Diseases, College of Medicine, Hallym University, Chuncheon 24252, Korea; nivirna07@hallym.ac.kr (K.-K.O.); dpfls3020@gmail.com (Y.-R.C.); phr.haripriya13@gmail.com (H.G.); vraja.ganesan@gmail.com (R.G.); satyapriya83@hallym.ac.kr (S.P.S.); lionbanana@hallym.ac.kr (S.-M.W.); jj_jeong@hallym.ac.kr (J.-J.J.); qlstn5549@gmail.com (S.-B.L.); qjarlf987@naver.com (M.-G.C.); ninetjd@naver.com (G.-H.K.); djkim@hallym.ac.kr (D.-J.K.)

**Keywords:** alcoholic liver disease (ALD), network pharmacology concept, PI3K-Akt signaling pathway, bacterium MRG-PMF-1, RELA, Icaritin

## Abstract

Alcoholic liver disease (ALD) is linked to a broad spectrum of diseases, including diabetes, hypertension, atherosclerosis, and even liver carcinoma. The ALD spectrum includes alcoholic fatty liver disease (AFLD), alcoholic hepatitis, and cirrhosis. Most recently, some reports demonstrated that the pathogenesis of ALD is strongly associated with metabolites of human microbiota. AFLD was the onset of disease among ALDs, the initial cause of which is alcohol consumption. Thus, we analyzed the significant metabolites of microbiota against AFLD via the network pharmacology concept. The metabolites from microbiota were retrieved by the gutMGene database; sequentially, AFLD targets were identified by public databases (DisGeNET, OMIM). The final targets were utilized for protein–protein interaction (PPI) networks and signaling pathway analyses. Then, we performed a molecular docking test (MDT) to verify the affinity between metabolite(s) and target(s) utilizing the Autodock 1.5.6 tool. From a holistic viewpoint, we integrated the relationships of microbiota-signaling pathways-targets-metabolites (MSTM) using the R Package. We identified the uppermost six key targets (TLR4, RELA, IL6, PPARG, COX-2, and CYP1A2) against AFLD. The PPI network analysis revealed that TLR4, RELA, IL6, PPARG, and COX-2 had equivalent degrees of value (4); however, CYP1A2 had no associations with the other targets. The bubble chart showed that the PI3K-Akt signaling pathway in nine signaling pathways might be the most significant mechanism with antagonistic functions in the treatment of AFLD. The MDT confirmed that Icaritin is a promising agent to bind stably to RELA (known as NF-Κb). In parallel, Bacterium MRG-PMF-1, the PI3K-Akt signaling pathway, RELA, and Icaritin were the most significant components against AFLD in MSTM networks. In conclusion, we showed that the Icaritin–RELA complex on the PI3K-Akt signaling pathway by bacterial MRG-PMF-1 might have promising therapeutic effects against AFLD, providing crucial evidence for further research.

## 1. Introduction

Alcoholic liver disease (ALD) has a causal relationship with the excess consumption of alcohol, which can be accumulated as excessive fats in the liver [1]. The liver is the primary organ that metabolizes alcohol, the damage of which can lead to the loss of liver function [2,3]. Excessive alcohol intake can cause alcoholic fatty liver disease (AFLD), hepatitis, fibrosis, cirrhosis, and even hepatocelluar carcinoma, which is the most severe liver damage associated with alcohol consumption [4].

Steatosis is an initial response to overdrinking, and it is expressed by the accumulation of fat in hepatocytes [5]. Steatosis can develop steatohepatitis, which gives birth to severe inflammatory liver injury [6].

Then, steatohepatitis can progress to fibrosis due to the deposition of an excessive extracellular matrix [7]. Fibrosis is defined as the inceptive stage of liver scarring, the aggravation of which can develope into cirrhosis [8]. To be specific, cirrhosis is a pathological stage exposed highly to hepatocellular carcinoma [9]. Thus, the progressive development of AFLD results in liver failure as an irreversible condition. Commonly, liver damage induced by alcohol may be recovered if abstinence is maintained persistently; as it were, alcohol cessation might be an optimal treatment against AFLD [10]. Currently, as an alternative therapy, metadoxine with potent alcohol-clearing effects from the blood has been utilized to treat alcohol-related liver disease, which is given through intravenous formulation [11].

In recent years, gut microbiota treatments have been considered as another alternative medicine that provides favorable therapeutic efficacy for fatty liver patients [12]. Previous studies demonstrated that diverse metabolites produced from gut microbiota might be agents to ameliorate fatty liver disorders, particularly by regulating the pharmacological mechanism(s) related to the immune system [13,14]. The polyphenolic metabolites converted by the gut microbiota might have beneficial effects on the host: antidiabetic, antiobesitic, and antiatherosclerotic effects [15].

Noticeably, flavonoids dampen reactive oxygen species (ROS), cholesterol synthesis, and apoptosis in hepatocytes [16]. The gut–liver axis is a crosstalk passage among the gut, its microbiota community, and the liver, generating the integration of signaling transduced by nutritional, hereditary, and environmental causes [17]. Therefore, we suggest that the exploration of metabolites from the gut microbiota can be a significant process to obtain promising therapeutic agents against ALD. In addition, the application of networks is an insightful frame for merging complex biological information such as protein–protein interactions (PPIs), metabolic networks, and functions of metabolites [18]. PPI network analysis is an effective methodology for drug repurposing, suggesting evidence about causal factors between targets that perform particular functions [19]. More importantly, we constructed microbiota-signaling pathways-targets-metabolites (MSTM) networks to identify the relative importance of each node. We conducted this study based on AFLD, because AFLD in ALD is the first alarming effect induced by excessive alcohol consumption. Therefore, this research aims to pioneer pharmacological mechanism(s) of gut microbiota metabolites in AFLD.

## 2. Hypothesis

The metabolites from the gut microbiota were identified by the gutMGene database, indicating that targets related directly to the metabolites were meta-analyzed by the Similarity Ensemble Approach (SEA) and SwissTargetPrediction (STP). The overlapping targets between gutMGene and AFLD associated with the meta-analyzed targets were considered the core targets against AFLD. We hypothesize that the core targets are important therapeutic elements to confirm the function on a key metabolite. Based on this hypothesis, we performed MDT to identify the most stable metabolite(s)–target(s) complex in a hub signaling pathway. Thus, we postulated that the gut microbiota-produced metabolite(s) bound most stably to a target on a hub signaling pathway are crucial indicators in treating AFLD.

## 3. Methods and Materials

The metabolites from the gut microbiota were retrieved by gutMGene (http://bio-annotation.cn/gutmgene/) (accessed on 21 May 2022). We utilized the Similarity Ensemble Approach (SEA) (https://sea.bkslab.org/) (accessed on 21 May 2022) [20] and SwissTargetPrediction (STP) (http://www.swisstargetprediction.ch/) (accessed on 22 May 2022) [21] to conduct the meta-analysis on the metabolites. AFLD targets were obtained by DisGeNET (https://www.disgenet.org/) (accessed on 23 May 2022) and OMIM (https://www.omim.org/) (accessed on 24 May 2022). The core targets were analyzed by STRING bioinformatics platform (https://string-db.org/) (accessed on 25 May 2022). We performed PPI network and MSTM analyses via R Package. The workflow of this study is as follows.

Step 1: Identification of metabolites from the gut microbiota via gutMGene.

Steps 2 and 3: Identification of targets related to the metabolites via the SEA and STP databases. The metabolites were input into the SEA and STP databases in SMILES format.

Step 4: Identification of AFLD targets via DisGeNET and OMIM.

Step 5: Identification of overlapping targets between Steps 2 and 3 and Step 4. The overlapping targets were identified by VENNY 2.1 (https://bioinfogp.cnb.csic.es/tools/venny/) (accessed on 25 May 2022) [22].

Step 6: Identification of core targets between Step 4 and targets via gutMGene.

Step 7: The construction of PPI networks from the core targets. Information on the target networks was identified by STRING (https://string-db.org/) (accessed on 25 May 2022) [23].

The PPI networks were visualized by the R package.

Step 8: The construction of a bubble chart to identify a hub-signaling pathway against AFLD. Based on the expressed gene ratio, the bubble chart was constructed by R Package.

Step 9: The first screening of the significant metabolites was based on Topological Polar Surface Area (TPSA) < 140 Å2 or Lipinski’s rule. SwissADME (http://www.swissadme.ch/) (accessed on 25 May 2022) was utilized to identify the physicochemical properties of drug-likeness about metabolite(s). The toxicological evaluation was confirmed by the ADMETlab 2.0 platform (accessed on 25 May 2022) [24].

Step 10: The second screening via MDT based on the threshold (<―6.0 kcal/mol) or the lowest Gibbs energy (the greatest negative value) of each metabolite. The metabolites were downloaded as. Sdf format from PubChem, which were converted into.pdb format via PyMOL. The .pdb format was converted into .pdbqt format to prepare for MDT via the AutoDockTools-1.5.6 tool. Then, the PDB IDs of the target(s) were identified by RCSB PDB (https://www.rcsb.org/) (accessed on 26 May 2022). MDT was performed to evaluate the affinity of metabolite(s)-target(s) by utilizing AutoDockTools-1.5.6.

The docking site was set up with a cubic box on the center: RELA (known as NF-Κb) (x = 23.285, y = 12.431, z = 86.636). The grid box size of the active site was set to x = 40 Å, y = 40 Å, and z = 40 Å. The identification of 2D binding interactions was performed by LigPlot+2.2. (https://www.ebi.ac.uk/thornton-srv/software/LigPlus/download2.html) (accessed on 27 May 2022) [25].

Step 11: Construction of the MSTM. The MSTM networks were visualized by R Package. The most significant components against AFLD were based on the degree of value.

The degree value of the microbiota, signaling pathway, target, or metabolite represents the edge numbers of the microbiota, signaling pathway, target, or metabolite in the MSTM network.

Taken together, the relationships of microbiota, signaling pathways, targets, and metabolites were constructed with Microsoft Excel and then incorporated into R Package to assemble the network of metabolites related to AFLD.

**Figure 1:**
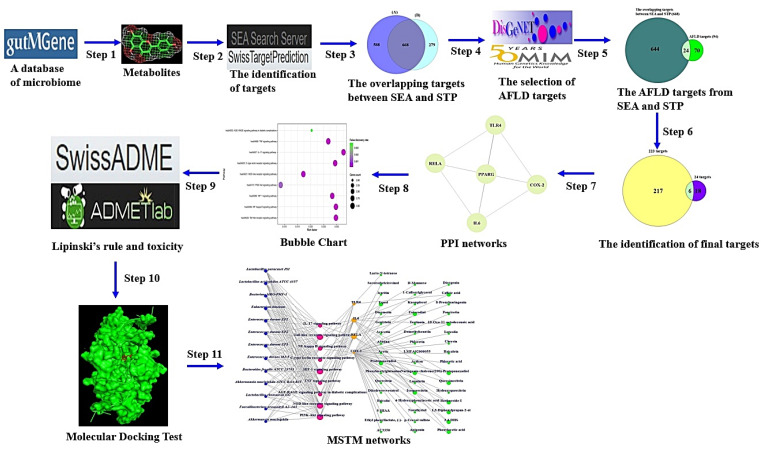
The workflow of this study.

The workflow of this study is displayed in Figure 1.

## 4. Results

**Figure 2:**
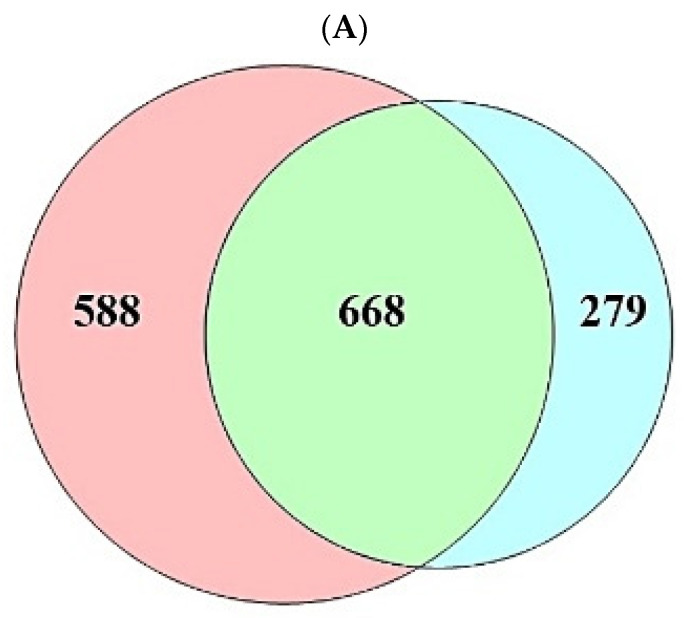

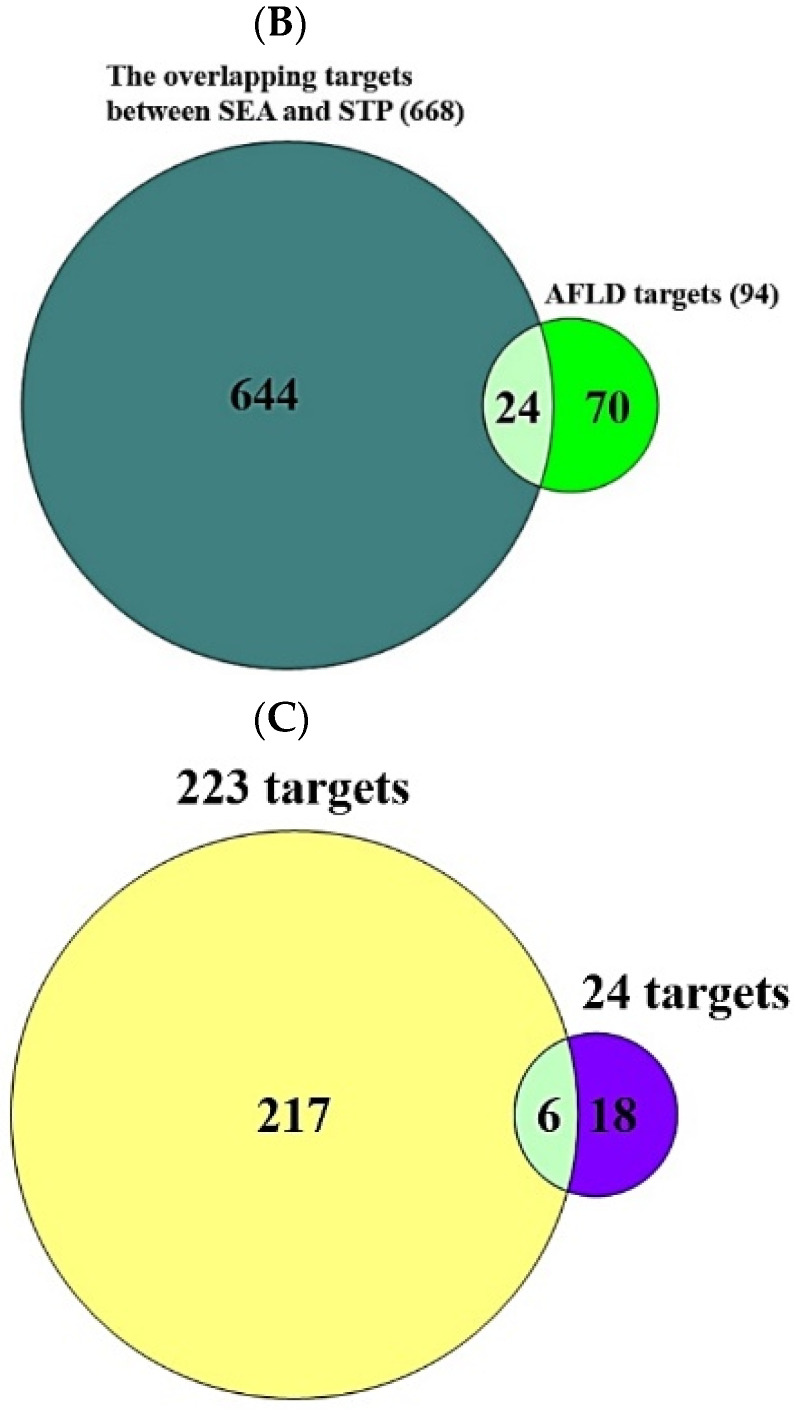
(**A**) The number of overlapping 668 targets between SEA (1256 targets) and STP (947 targets) database. (**B**) The number of overlapping 24 targets between the 668 targets and AFLD-related targets (94 targets). (**C**) The number of the final overlapping 6 targets between the 24 targets and gut human targets (223 targets).

A total of 208 (metabolites) were retrieved from the gutMGene database, targets of which were identified by the SEA (1256) and STP (947) databases (Supplementary Table S1). The number of 668 overlapping targets was identified by the SEA (1256) and STP (947) databases (Figure 2A) (Supplementary Table S1). The overlapping targets (24) were obtained between the overlapping 668 targets and the targets (94) related directly to AFLD, and then the final overlapping targets (6) were identified between targets (223) from gutMGene and 24 targets (Figure 2B,C) (Supplementary Table S1).

**Figure 3:**
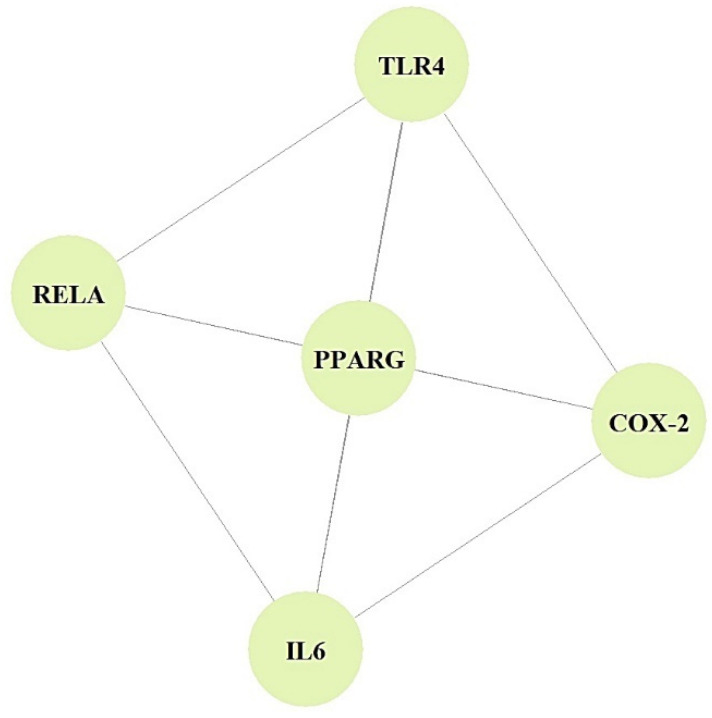
PPI networks (5 nodes, 10 edges).

In the PPI networks, CYP1A2 did not interact with the other five targets (PPARA, TLR4, COX-2, IL6, and RELA) and comprised five nodes and 10 edges (Figure 3).

**Figure 4:**
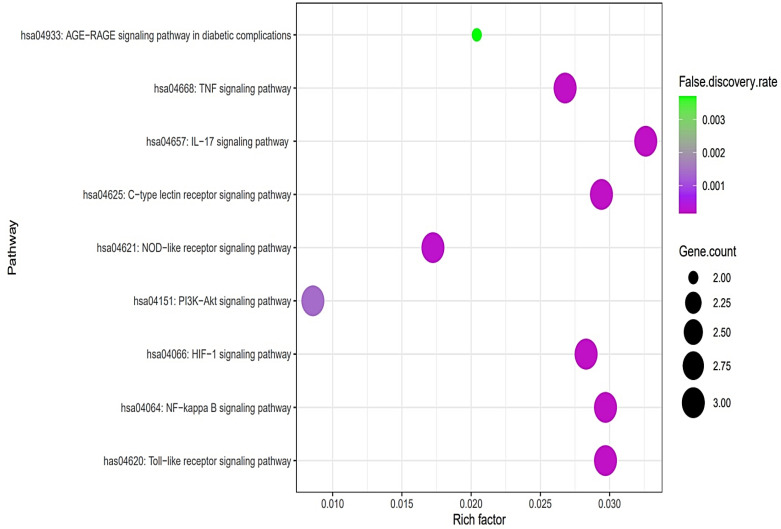
A bubble chart of nine signaling pathways associated with AFLD.

The PPI networks suggested that the five core targets had the same degree value (5). Next, the results of a bubble chart indicated that four overlapping targets were significantly enriched in nine signaling pathways (false discovery rate < 0.05) (Figure 4).

**Figure 5:**
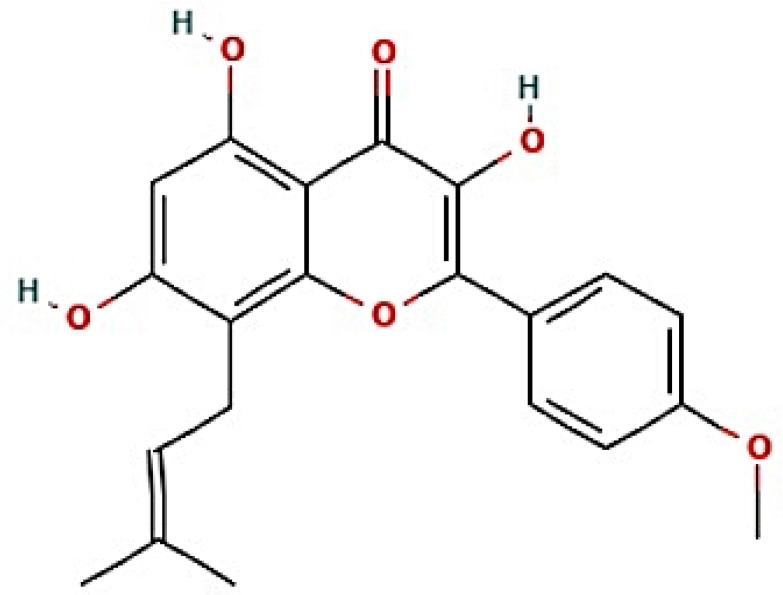
The structure of Icaritin.

The nine signaling pathways were directly associated with the progression of AFLD, suggesting that these signaling pathways might be the modes of action against AFLD. Additionally, RELA (the key target) in the MSTM network was directly enriched in all nine signaling pathways by the PI3K-Akt signaling pathway as a hub signaling pathway against AFLD. Three metabolites (Icaritin, lacto-N-tetraose, and quercimeritrin) which bound stably to the PI3K-Akt signaling pathway were identified; however, we removed two metabolites (lacto-N-tetraose and quercimeritrin) due to a violation of Lipinski’s rule. Thus, we confirmed that Icaritin (Figure 5) is a promising metabolite based on physicochemical properties and toxicity via the SwissADME and ADMETlab platforms (Table 1).

**Figure 6:**
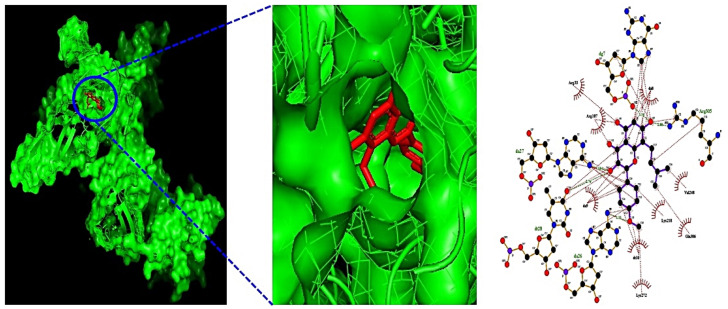
The 3D and 2D diagram of Icaritin (PubChem ID: 5318980) on RELA (PDB ID: 2O61).

**Figure 7:**
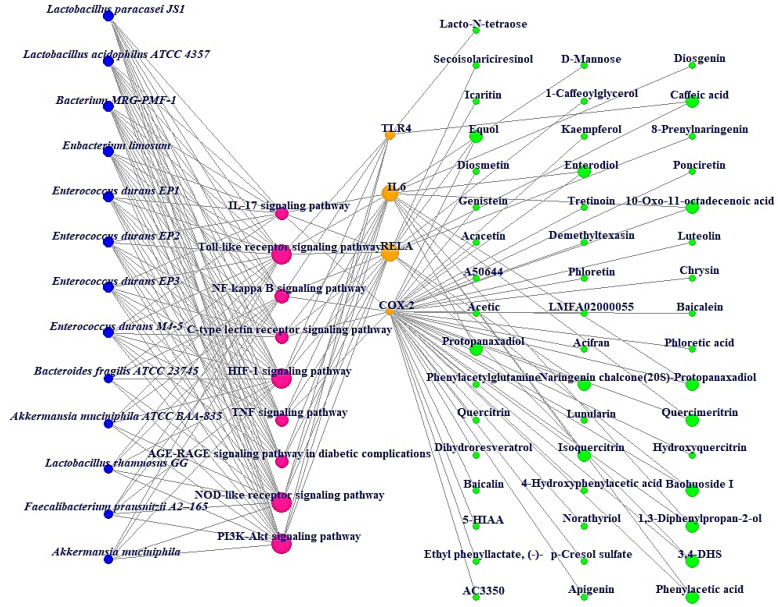
MSTM networks (75 nodes and 181 edges). Blue circle: gut microbiota; red circle: signaling pathway; orange circle: target; green circle: metabolite.

Additionally, MDT demonstrated that Icaritin (Gibbs energy: −10.0 kcal/mol) bound stably to RELA, which is associated with the PI3K-Akt signaling pathway (Table 2) (Figure 6). MSTM network analysis showed that Bacterium MRG-PMF-1, the PI3K-Akt signaling pathway, RELA, and Icaritin were the most significant components in treating AFLD (Figure 7) (Table 3).

## 5. Discussion

The results of the bubble chart suggested that the nine signaling pathways might be therapeutic mechanisms that ameliorate AFLD. The relationships of the nine signaling pathways with AFLD are concisely expounded as follows. (1) AGE-RAGE signaling pathway in diabetic complications: The AGE-RAGE interaction accelerates the accumulation of fat in the liver, which causes inflammation, fibrosis, and other disorders of fatty liver disease [26]. It follows that the inhibition of the AGE-RAGE signaling pathway might be a therapeutic strategy against AFLD. (2) Tumor necrosis factor (TNF) signaling pathway: TNFα can stimulate the liver inflammation that generates liver fibrosis; however, the function of TNFα in liver disease has not been completely elucidated [27]. (3) Interleukin 17 (IL-17) signaling pathway: IL-17A significantly regulates alcohol-induced hepatic steatosis linked directly to inflammatory responses [28]. (4) C-type lectin receptor (CLR) signaling pathway: C-type lectin receptors such as Dectin-1, Dectin-2, and Dectin-3 are signal receptors that recognize pathogen-associated molecular patterns (PAMPs); in particular, Dectin-1 is overexpressed in hepatic fibrosis [29]. (5) NOD-like receptor signaling pathway: NOD-like receptor proteins are known to control innate immune responses against cellular damage [30]. (6) PI3K-Akt signaling pathway: The inhibition of the PI3K-Akt signaling pathway drives anti-inflammatory effects by inhibiting NF-κB [31]. (7) Hypoxia-inducible factor-1 (HIF-1) signaling pathway: HIF-1 aggravates lipid droplet buildup, which can boost the metabolism of fatty acids [32]. (8) NF-κB signaling pathway: NF-κB effectors stimulate lipogenesis in hepatocytes, which is activated in AFLD [33,34]. (9) Toll-like receptor (TLR) signaling pathway: TLRs are implicated in hepatic inflammation, and an understanding of the mechanism might be manifested as a new therapeutic target [35].

The MSTM network suggested that the components directly related to therapeutic effects on AFLD consist of 13 microbiota, 9 signaling pathways, 4 targets, and 49 metabolites. Based on their degrees of value, Bacterium MRG-PMF-1 (97), PI3K-Akt signaling pathway (12), RELA (9), and Icaritin (1) were the most significant elements for alleviating AFLD.

Additionally, Lactobacillus paracasei JS1 and Lactobacillus acidophilus ATCC 4357 with the same degree of value as Bacterium MRG-PMF-1 can produce equol with anti-inflammatory effects and function as alleviators of digestive disorders, respectively [36,37,38]. Notably, a study demonstrated that PI3K/Akt inhibitors might be a therapeutic strategy for the alleviation of fatty liver damage induced by ethanol, suppressing autophagic degradation of lipid bodies [39]. RELA (known as NF-κB) has a characteristic relevance in the inflammatory pathways such as alcoholic-driven liver stress [33,40]. Icaritin is a metabolite that is produced via Bacterium MRG-PMF-1, and has potent antioxidant and anti-inflammatory properties to prevent liver damage [41,42].

From a systemic viewpoint via network pharmacology, this study initially elaborates on the key microbiota, critical signaling pathways, significant targets, and crucial metabolites against AFLD. Comprehensively, we have analyzed the uppermost components by integrating networks to expound the therapeutic elements against AFLD. Given the limitations of the database, the indicated four factors are based on data mining; however, the MSTM network represents crosstalk between the host and microbiota. Finally, this study requires further clinical trials to verify its therapeutic benefits based on scientific evidence from this research.

## 6. Conclusions

In summary, this study provides promising components for treating AFLD via microbiota-based analysis of the network pharmacology concept. In this analysis, we revealed that Bacterium MRG-PMF-1, which produces Icaritin, bound stably to RELA to inhibit PI3K-Akt signaling pathway. Our findings also contribute to revealing the therapeutic value of metabolites from microbiota, which needs further validation in clinical trials.

## Figures and Tables

**Table 1:** The toxicity parameters of Icaritin.

Parameters	Metabolite
	Icaritin
hERG (hERG blockers)	Non-blockers
Rat oral Acute Toxicity	Negative
Carcinogenecity	Negative
Eye corrosion	Negative
Respiratory toxicity	Negative
LD50 (LD50 of acute toxicity)	5.914 mg/kg

**Table 2:** The binding energy and amino acid residues interacted with Icaritin–RELA (known as NF-Κb) complex.

				Grid Box		Hydrogen Bond Interactions	Hydrophobic Interactions
Protein	Ligand	PubChem ID	Binding Energy (kcal/mol)	Center	Dimension	Amino Acid Residue	Amino Acid Residue
RELA (PDB ID: 2O61)	Icaritin	5318980	−10.0	x = 15.616	size_x = 40	Arg305	Val248, Lys218, Gln306
				y = −22.641	size_y = 40		Lys272, Arg33, Arg187
				z = −18.824	size_z = 40		
	Baohuoside I	5488822	−9.7	x = 15.616	size_x = 40	Arg1011	Glu222, Lys221, Gln241
				y = −22.641	size_y = 40		
				z = −18.824	size_z = 40		
	8-Prenylnaringenin	480764	−9.5	x = 15.616	size_x = 40	Lys272, Lys218, Arg187	Gln306, Arg246, Phe307
				y = −22.641	size_y = 40		Val248, Arg305, Asp217
				z = −18.824	size_z = 40		
	Equol	91469	−8.4	x = 15.616	size_x = 40	Asn186, Arg305, Gln306	Ala192, Asp217, Lys218
				y = −22.641	size_y = 40		Val248, Phe307, Arg187
				z = −18.824	size_z = 40		
	Secoisolariciresinol	65373	−8.4	x = 15.616	size_x = 40	Arg305, Gln306	Arg33, Arg187, Lys218
				y = −22.641	size_y = 40		Arg246, Phe307, Gln247
				z = −18.824	size_z = 40		Val248
	Naringenin chalcone	5280960	−8.3	x = 15.616	size_x = 40	Arg33, Asn186	Phe307, Gln306, Arg305
				y = −22.641	size_y = 40		Arg187
				z = −18.824	size_z = 40		
	3,4-Dihydroxy-trans-stilbene	10176710	−7.5	x = 15.616	size_x = 40	Gln306	Phe307, Val248, Lys218
				y = −22.641	size_y = 40		Asn186, Ala192, Asp217
				z = −18.824	size_z = 40		Arg305
	2,3-Dihydroxypropyl (E)-3-(3,4-dihydroxyphenyl)prop-2-enoate	5315606	−7.2	x = 15.616	size_x = 40	Arg246	Gln306, Lys272, Lys241
				y = −22.641	size_y = 40		Phe307
				z = −18.824	size_z = 40		
	Caffeic acid	689043	−6.6	x = 15.616	size_x = 40	Gln306, Arg305, Arg33	Val248, Phe307
				y = −22.641	size_y = 40		
				z = −18.824	size_z = 40		

**Table 3:** The degree of value on the MSTM network.

**No.**	**Microbiota**	**Degree of Value**
1	Bacterium MRG-PMF-1	97
2	Lactobacillus paracasei JS1	97
3	Lactobacillus acidophilus ATCC 4357	97
4	Eubacterium limosum	97
5	Enterococcus durans EP1	93
6	Enterococcus durans EP2	93
7	Enterococcus durans EP3	93
8	Enterococcus durans M4-5	93
9	Bacteroides fragilis ATCC 23745	82
10	Akkermansia muciniphila ATCC BAA-835	82
11	Lactobacillus rhamnosus GG	82
12	Faecalibacterium prausnitzii A2<U+2013>165	82
13	Akkermansia muciniphila	82
**No.**	**Signaling pathways**	**Degree of Value**
1	hsa04151: PI3K-Akt signaling pathway	13
2	hsa04621: NOD-like receptor signaling pathway	13
3	hsa04066: HIF-1 signaling pathway	13
4	has04620: Toll-like receptor signaling pathway	13
5	hsa04064: NF-kappa B signaling pathway	9
6	hsa04657:IL-17 signaling pathway	8
7	hsa04625: C-type lectin receptor signaling pathway	8
8	hsa04668: TNF signaling pathway	8
9	hsa04933: AGE-RAGE signaling pathway in diabetic complications	8
**No.**	**Targets**	**Degree of Value**
1	RELA	9
2	IL6	8
3	TLR4	5
4	COX-2	4
**No.**	**Metabolites**	**Degree of Value**
1	Phenylacetic acid	2
2	3,4-DHS	2
3	1,3-Diphenylpropan-2-ol	2
4	Baohuoside I	2
5	Isoquercitrin	2
6	Quercimeritrin	2
7	Naringenin chalcone	2
8	(20S)-Protopanaxadiol	2
9	Protopanaxadiol	2
10	10-Oxo-11-octadecenoic acid	2
11	Enterodiol	2
12	Equol	2
13	Caffeic acid	2
14	AC3350	1
15	Apigenin	1
16	Ethyl phenyllactate, (-)-	1
17	p-Cresol sulfate	1
18	5-HIAA	1
19	Norathyriol	1
20	Baicalin	1
21	4-Hydroxyphenylacetic acid	1
22	Dihydroresveratrol	1
23	Hydroxyquercitrin	1
24	Quercitrin	1
25	Lunularin	1
26	Phenylacetylglutamine	1
27	Acifran	1
28	Phloretic acid	1
29	Acetic	1
30	LMFA02000055	1
31	Baicalein	1
32	A50644	1
33	Phloretin	1
34	Chrysin	1
35	Acacetin	1
36	Demethyltexasin	1
37	Luteolin	1
38	Genistein	1
39	Tretinoin	1
40	Diosmetin	1
41	Ponciretin	1
42	Kaempferol	1
43	8-Prenylnaringenin	1
44	Icaritin	1
45	1-Caffeoylglycerol	1
46	Secoisolariciresinol	1
47	D-Mannose	1
48	Diosgenin	1
49	Lacto-N-tetraose	1

## Data Availability

All data generated or analyzed during this study are included in this published article (and its Supplementary Information Files).

## Supplementary Materials

The following supporting information can be downloaded at: https://www.mdpi.com/article/10.3390/cimb44070224/s1, Table S1: The number of 1256 targets from SEA; the number of 948 targets from STP; the number of 668 overlapping targets between SEA and STP; the number of 94 targets related to AFLD targets; the number of 24 targets between two databases (SEA and STP) and AFLD targets; the number of 6 final targets.

## Abbreviations

ALD	Alcoholic Liver Disease
AFLD	Alcoholic Fatty Liver Disease
CLR	C-type lectin receptor
HIF-1	Hypoxia Inducible Factor-1
IL-17	Interleukin 17
MDT	Molecular Docking Test
MSTM	Microbiota-Signaling pathways-Targets-Metabolites
PAMPs	Pathogen- Associated Molecular Patterns
PPI	Protein–Protein Interaction
SEA	Similarity Ensemble Approach
STP	SwissTargetPrediction
TLR	Toll-like receptor
TNF	Tumor Necrosis Factor
TPSA	Topological Polar Surface Area
